# Low Intensity Vibrations Augment Mesenchymal Stem Cell Proliferation and Differentiation Capacity during *in vitro* Expansion

**DOI:** 10.1038/s41598-020-66055-0

**Published:** 2020-06-10

**Authors:** Guniz Bas, Stacie Loisate, Stephanie F. Hudon, Kali Woods, Eric J. Hayden, Xinzhu Pu, Richard Beard, Julia T. Oxford, Gunes Uzer

**Affiliations:** 1 Mechanical and Biomedical Engineering, Boise State University, Boise, USA; 2 Biomolecular Sciences Graduate Program, Boise State University, Boise, USA; 3 Biological Sciences, Boise State University, Boise, USA; 4 Biomolecular Research Center, Boise State University, Boise, USA

**Keywords:** Biomedical engineering, Stem cells

## Abstract

A primary component of exercise, mechanical signals, when applied in the form of low intensity vibration (LIV), increases mesenchymal stem cell (MSC) osteogenesis and proliferation. While it is generally accepted that exercise effectively combats the deleterious effects of aging in the musculoskeletal system, how long-term exercise affects stem cell aging, which is typified by reduced proliferative and differentiative capacity, is not well explored. As a first step in understanding the effect of long-term application of mechanical signals on stem cell function, we investigated the effect of LIV during *in vitro* expansion of MSCs. Primary MSCs were subjected to either a control or to a twice-daily LIV regimen for up to sixty cell passages (P60) under *in vitro* cell expansion conditions. LIV effects were assessed at both early passage (EP) and late passage (LP). At the end of the experiment, P60 cultures exposed to LIV maintained a 28% increase of cell doubling and a 39% reduction in senescence-associated β-galactosidase activity (p < 0.01) but no changes in telomere lengths and p16^INK4a^ levels were observed. Prolonged culture-associated decreases in osteogenic and adipogenic capacity were partially protected by LIV in both EP and LP groups (p < 0.05). Mass spectroscopy of late passage MSC indicated a synergistic decrease of actin and microtubule cytoskeleton-associated proteins in both control and LIV groups while LIV induced a recovery of proteins associated with oxidative reductase activity. In summary, our findings show that the application of long-term mechanical challenge (+LIV) during *in vitro* expansion of MSCs for sixty passages significantly alters MSC proliferation, differentiation and structure. This suggests LIV as a potential tool to investigate the role of physical activity during aging.

## Introduction

Mesenchymal stem cells (MSC) found in adult musculoskeletal tissues make up approximately 0.01 to 0.001% of all cells^1^ and are responsible for maintaining tissue turnover and, in case of injury, provide necessary cell populations by first proliferating and then differentiating into specific cell types. In bone, MSC is the common progenitor for osteoblasts, osteocytes and adipocytes^2–4^. During bone injury, for example, an immune response signals MSCs to proliferate and populate the fibrin matrix. These localized MSCs first differentiate into chondrocytes and subsequently become osteoblasts to initiate the bone remodeling process^5^. During habitual loading, MSCs also provide necessary osteoblast populations to facilitate bone modeling when bones are loaded beyond the physiological “sweet-spot” (~2000–3000 micro-strain), thus resulting in a net increase of bone formation^6^.

The aging phenotype of MSCs is associated with decreased proliferative and differentiative capacity^7,8^, which delays healing and impairs the repair of wear and tear in musculoskeletal tissues. This contributes to the steady degeneration associated with aging as well as increased injury risk and fractures^9^. Such impairment of MSC function also poses a problem for regenerative medicine and tissue engineering approaches that primarily rely on allogenic cell populations from aged individuals^10^. Therefore, there is a critical need to improve MSC functionality during aging.

Exercise is one of the most commonly prescribed activities to combat the effects of aging. Physically active adults, for example, are likely to have reduced risk of hip as well as vertebral fracture, along with increased skeletal muscle mass, strength, power, and intrinsic neuromuscular activation^11^. Despite the benefits of exercise, there are only a handful of studies that have investigated the role of exercise on MSC function. One study examined the effects of a 3-month-long daily 15 m/min treadmill activity on MSCs from adult 6-month-old female rats. Compared to neonatal MSCs (day 2), decreased osteogenic capacity in the sedentary adult group was partially recovered following exercise^12^. Another study investigated the effect of a 4-month-long daily ladder climbing regimen, started at 17-months of age, on MSCs isolated from rats: compared to adult MSCs from 5-month-old rats, aged MSCs showed decreased osteogenesis which was partially restored by 4-month-long exercise intervention^13^. While these studies highlight the benefits of exercise at the level of MSC, mechanical signals that are produced during exercise are accompanied by changes in cardiorespiratory fitness and caloric expenditure at a systemic level that may confound these outcomes^14,15^. Thus, there is a knowledge gap with respect to how isolated mechanical signals affect MSC function when applied long-term.

MSCs in bone exist in a mechanically rich environment. Measurement of daily loading history in weight-bearing tibia for different species demonstrated that while bones experience a small number of high magnitude (~2000–3000 microstrain), low-frequency events (1–2 Hz), they are bombarded by hundreds of thousands of smaller magnitude signals (<10 microstrain) that are the result of high-frequency muscle contractions (10–50 Hz)^16^. These low-magnitude mechanical events in the skeleton decrease with age-related muscle weakness or disuse^17^, providing a connection between muscle and bone loss. Indeed, exogenous application of low-magnitude mechanical signals, by using low-intensity vibration (LIV) platforms, provides a physiologic mechanical input to preserve musculoskeletal competence. In women with osteoporosis or young women with low bone mineral density, LIV has been shown to improve bone and muscle indices as well as balance^18,19^. Animal studies demonstrate that exogenously delivered mechanical stimulation is sufficient to increase trabecular bone volume^20^, increase bone stiffness and strength^21^, and to slow the bone loss caused by disuse^22^.

At the cellular scale, our group has reported that LIV increases the phosphorylation of Focal Adhesion Kinase (FAK) at Tyrosine 397 and Akt at Serine 473 residues, resulting in increased GTP bound RhoA levels and robust F-actin bundling^23^. The effects of LIV are additive, with a second bout of LIV augmenting FAK phosphorylation and F-actin contractility due to either mechanical strain or RhoA activating agents like Lysophosphatidic Acid^23^. When LIV is applied twice daily for seven days, mRNA expression panels show significant increases in F-actin modulatory genes in LIV groups when compared to non-LIV controls, including RhoA stimulator *ARHFGEF11* (Rho Guanine Nucleotide Exchange Factor 11) and Arp2/3 complex regulator *WAS* (Wiskott-Aldrich syndrome)^24^. These positive effects of LIV on actomyosin contractility translate into increased focal adhesions^23^ as well as increased stiffness of F-actin struts^25^. Concomitant to changes in cellular structure, LIV activates nuclear effectors such as βcatenin and its subsequent accumulation in the nucleus^26^. Functionally, these changes lead to the improvement of osteogenic and proliferative capacity in MSCs^24,27^.

**Figure 1:**
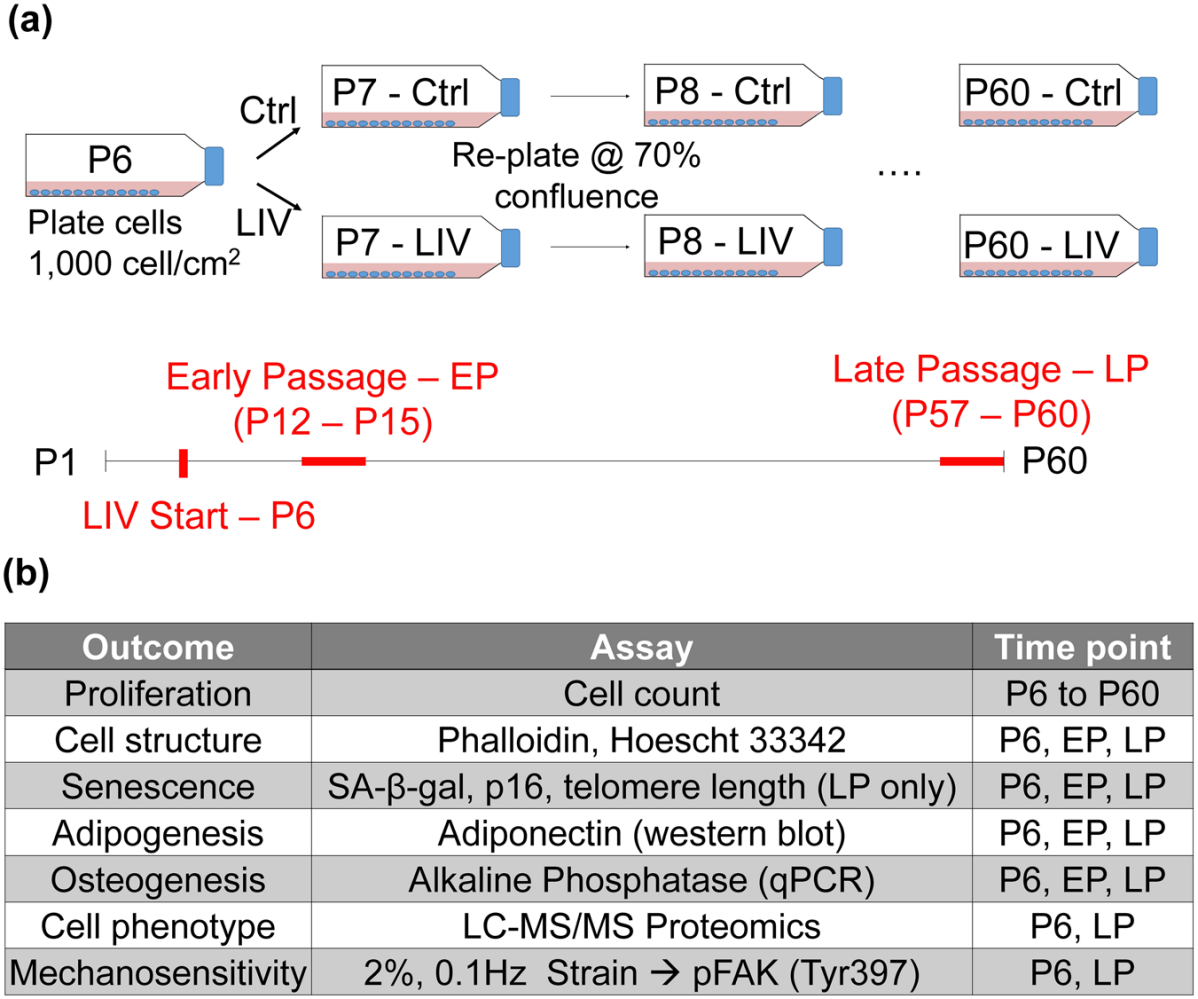
*In vitro* MSC expansion model and experimental design. (**a**) MSCs at passage 6 (P6) were serially passaged until P60. Experimental groups were subjected to twice-daily Low Intensity Vibration (LIV) regimen (0.7 g, 90 Hz, 20 minutes) outside of the incubator. While control MSCs were taken out of the incubator for 20 minutes twice daily, they were not vibrated. Each day, flasks were observed for confluence and when either one of the groups reached 70% confluence (non-contact), both groups were trypsinized, counted and re-plated at the density of 1000 cell/cm^2^. This protocol was repeated until passage 60 (P60). (**b**) Data collected between P12 and P15 were designated as early passage (EP) and data collected P57 and P60 were designated as late passage (LP). All assays were performed 24 h after plating with no LIV applied within the 24 h period. EP and LP samples were compared to P6 samples in separate, independent experiments with the exception of immunostaining where samples were fixed at different times but were stained and analyzed together.

Long-term serial passaging of cells are commonly used as a model where culture-expanded cells show reduced proliferative capacity and differentiation potential with a higher susceptibility to cell senescence^28^. While not entirely consistent with chronological aging, *in vitro* models have been used to capture aspects of aging *in vitro*^29–31^. As aging is a multifaceted condition with complex interactions, controllable and simplified conditions of *in vitro* culture provides an ideal model to study the isolated effects of long-term LIV application on MSC function. Therefore, in this study we tested the hypothesis that daily application of LIV will improve proliferation and differentiation capacity of MSCs during prolonged cell culture. We have used a long-term serial passaging with and without daily application of LIV (Fig. 1a). Changes in MSC proliferation, differentiation, senescence markers, mechanosensitivity and proteome were compared using immunofluorescence, qPCR, western blotting and liquid chromatography-tandem mass spectrometry (LC-MS/MS) (Fig. 1b).

## Results

### Low intensity vibration (LIV) mitigates reduced proliferation during *in vitro* expansion of MSCs

**Figure 2:**
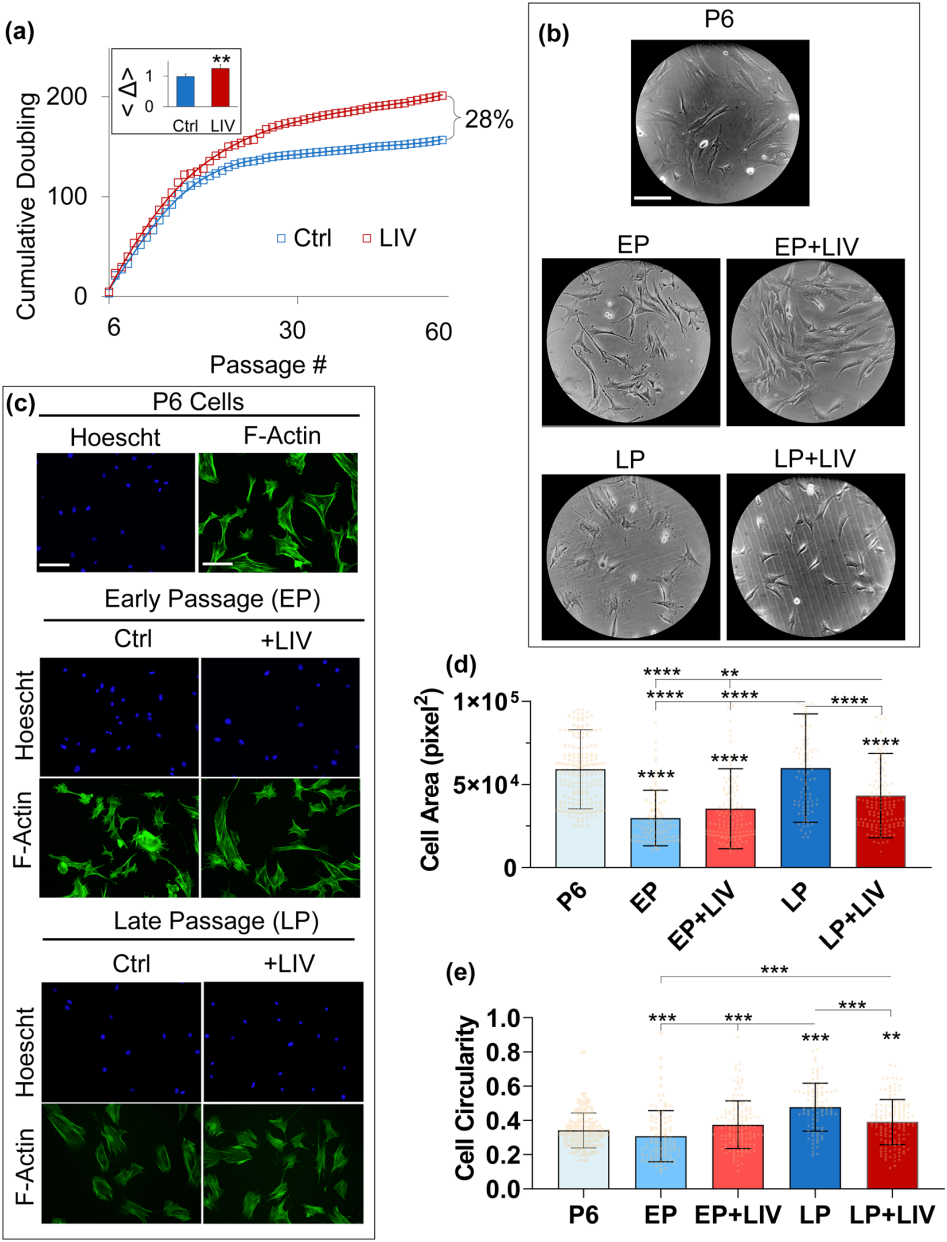
LIV increases proliferation and decreases cell size. (**a**) Long term-LIV resulted in a consistent improvement of proliferation over 30+ weeks, adding up to an 28% cumulative difference of cell doubling at passage 60 (P60). On average, +LIV group had a 27.7% (p < 0.01) increased cell doubling when compared to non-LIV control (Δ, inset on top left). (**b**) Cell morphology at EP and LP time points showed that non-LIV controls exhibited a more circular while +LIV groups were more spindle like and elongated. (**c**) Fluorescent labeling of cell nuclei (Hoechst 33342) and F-actin (phalloidin) were performed to quantify cell and nuclear morphology. (**d**) Cell area of the P6 group was 5.90×104 px^2^, areas of the EP and EP + LIV groups were 2.97×104 px2 and 3.53×104 px^2^, showing 50% and 40% reduced cell area, respectively (p < 0.0001). The cell area of the LP group was 5.97×104 px2 and was not different than the P6 group. Cell area of the LP group remained 51% and 41% larger compared to both the EP and EP + LIV groups (p < 0.0001). Cell area of The LP + LIV group was 4.32×104 px^2^, a 27% and 28% decrease when compared to the P6 and LP groups, respectively (p < 0.0001). Cell area of The LP + LIV group also remained 32% (p < 0.0001) and 19% (p < 0.01) elevated when compared to the EP and EP + LIV groups, respectively. (**d**) Average circularity of P6 control was 0.34 where “1” represents a perfect circle and “0” represents a line. EP and EP + LIV showed no changes in cell circularity when compared to P6. Cell circularity of non-LIV LP controls was 0.47, a 38.2% increase when compared to the P6 group (p < 0.0001). Compared to LP, average cell circularity of LP + LIV group was 0.39, a 22% decrease when compared to non-vibrated LP MSCs (p < 0.001). Circularity of LP + LIV remained 13% more compared to P6 MSCs (p < 0.01). Results are presented as mean ± STD. Scale bar: 100 µm. n = 3/grp (**b**,**c**), non-parametric two-tailed Mann-Whitney U-test (2a) or Kruskal-Wallis test followed by Dunn’s multiple comparison test (2d-e). p < 0.05, **p < 0.01, ***p < 0.001, ****p < 0.0001 against control and each other.

To test the long-term effect of LIV during *in vitro* expansion, primary MSCs were subjected to either a control or to a twice-daily LIV regimen (Fig. 1a). Shown in Fig. 2a, at passage 20 (P20), proliferation rates of non-LIV control groups began to slow down; in contrast, cells exposed to LIV ( + LIV) maintained a higher proliferation rate, increasing the cumulative cell doubling by 28% at P60. When cell doubling differences between the non-LIV and LIV groups in each cell passage were averaged (designated as Δ), + LIV group had a 27.7% increase in average cell doubling when compared to non-LIV control (p < 0.01, inset on top left).

### LIV decreases cell and nuclear size during prolonged cell passaging

*In vitro* expansion of MSCs has been shown to be associated with increased cell roundness^32^. Shown in Fig. 2b, visual comparison of gross cell morphology of P6 MSCs at early passage (EP) and late passage (LP) time points showed that non-LIV controls exhibited a more pronounced cell spreading. P6 MSCs in comparison were more spindle like and elongated in shape. +LIV groups on the other hand were similar in morphology to P6, appearing more spindle like. As shown in Fig. 2c, to quantify these characteristics, we measured cell and nuclear morphology via fluorescent labeling of cell nuclei (Hoechst 33342) and F-actin (phalloidin). Shown in Fig. 2d, when compared to the cell area of the P6 group (5.90×10^4^ px^2^), areas of the EP and EP + LIV groups were 2.97×10^4^ px^2^ and 3.53×10^4^ px^2^, showing 50% and 40% reduced cell area, respectively (p < 0.0001). The cell area of the LP group was 5.97×10^4^ px^2^ and was not different than the P6 group while remained 51% and 41% larger compared to both the EP and EP + LIV groups, respectively (p < 0.0001). Cell area of The LP + LIV group was 4.32×10^4^ px^2^, a 27% and 28% decrease when compared to the P6 and LP groups (p < 0.0001). Cell area of The LP + LIV group remained 32% (p < 0.0001) and 19% (p < 0.01) elevated when compared to the EP and EP + LIV groups, respectively. Increased cell circularity has been reported during long-term cell culture^32^. Average circularity of P6 control was 0.34 where “1” represents a perfect circle and “0” represents a line. Shown in Fig. 2d, EP and EP + LIV showed no changes in cell circularity when compared to P6. Cell circularity of non-LIV LP controls was 0.47, a 38.2% increase when compared to the P6 group (p < 0.0001). Compared to LP, average cell circularity of LP + LIV group was 0.39, a 22% decrease when compared to non-vibrated LP MSCs (p < 0.001). Circularity of LP + LIV remained 13% more compared to P6 MSCs (p < 0.01). As depicted in Fig.S1a nuclear areas of the EP, EP + LIV and the LP groups were not significantly altered when compared to the nuclear area of P6 MSCs which was 2.54×10^3^ px^2^. The nuclear area of the LP + LIV group was 1.77×10^3^ px^2^, exhibiting a 40% decrease compared to the P6 group. Nuclear area of the LP + LIV group also remained significantly lower than the EP, EP + LIV and the LP groups (p < 0.0001). Shown in Fig. S1b, no change in nuclear circularity was observed.

### *In vitro* expansion and LIV alters β-galactosidase activity but not p16 levels and telomere length in MSCs

**Figure 3:**
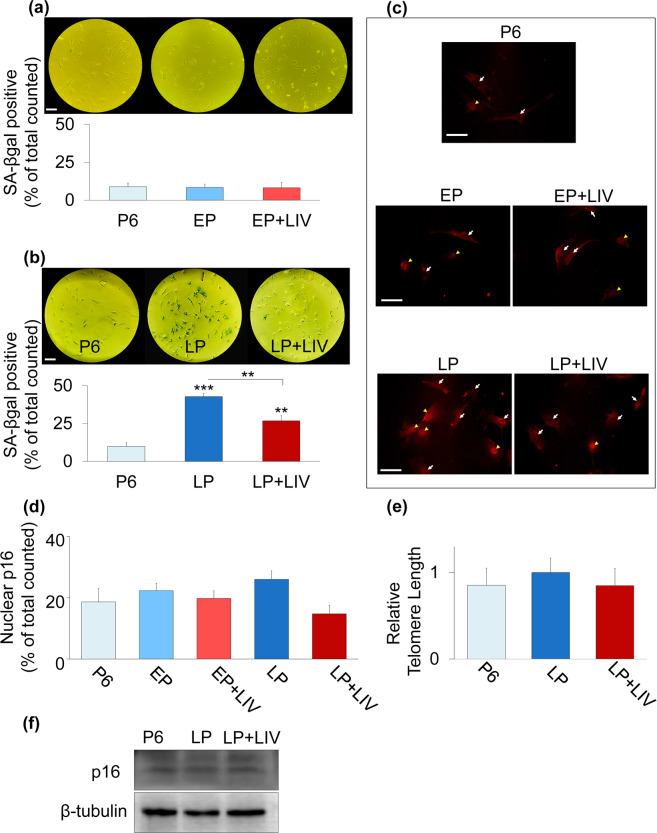
LIV *In vitro* expansion and LIV alters β-galactosidase activity but not p16 levels and telomere length in MSCs. (**a**) SA-βgal experiments at EP and LP time points were run, stained and analyzed independently. 8% of P6 MSCs were identified as SA-βgal positive and neither the EP nor EP + LIV groups showed any significant change compared to P6. (**b**) The P6 group was 9.8% SA-βgal positive, 42.8% of cells in LP group were identified as SA-βgal positive (P < 0.001) and this was decreased to 26.8% in LP + LIV group. Percentage of SA-βgal positive cells in +LIV group was significantly lower when compared to LP (p < 0.01) but remained significantly elevated compared to P6 (p < 0.01). (**c**) Immunolabeling against p16 was visible in cytoplasm (white arrows) and in the nuclei (yellow arrowheads). (**d**) Quantification of p16 positive nuclei showed no differences between groups. (**e**) There was no measurable difference between telomere lengths of P6, LP and LP + LIV groups and (**f**) western blotting of p16 protein also showed no difference between P6, LP and LP + LIV MSCs. Full-length blots are presented in Supplementary Fig.S2. Scale bar: 100 µm. n = 3/grp, group comparisons were made using one-way ANOVA followed by a Newman-Keuls post-hoc test (**a**,**b**,**e**,**f**) or Kruskal-Wallis test followed by Dunn’s multiple comparison test (**d**). p < 0.05, **p < 0.01, ***p < 0.001, against control and each other.

We next probed senescence markers by Senescence Associated β-galactosidase (SA-βgal) activity, telomere length measurements and p16^INK4A^ (referred as p16). SA-βgal experiments at EP and LP timepoints were run, stained and analyzed independently. As shown in Fig. 3a, only 8% of P6 MSCs were identified as SA-βgal positive and neither the EP nor EP + LIV groups showed any significant change compared to P6 controls. At LP time point, in contrast to 9.8% SA-βgal positive cells in P6 MSCs, 42.8% of cells in the non-LIV LP group were identified as SA-βgal positive (P < 0.001) and this was decreased to 26.8% in the LP + LIV group (Fig. 3b). The percentage of SA-βgal positive cells in the LP + LIV group was significantly lower when compared to the LP group (p < 0.01) but remained significantly elevated compared to P6 controls (p < 0.01). Next we probed another senescence associated marker p16 via immunofluorescence and western blotting^32^. As shown in Fig. 3c p16 staining was visible in cytoplasm (white arrows) and in the nuclei (yellow arrowhead). Quantification of p16 positive nuclei showed no differences (Fig. 3d). Additionally, we probed telomere length in LP groups as increased telomere attrition has been associated with a senescent phenotype^33^. Shown in Fig. 3e, there was no measurable difference between telomere lengths of P6, LP and LP + LIV groups. Similarly, western blotting of p16 protein also showed no difference between P6, LP and LP + LIV MSCs (Fig. 3f).

### LIV improves MSC differentiation potential into osteogenic and adipogenic lineages

To understand what preserved proliferative ability means for MSC fate, we quantified the osteogenic and the adipogenic capacity of EP, EP + LIV, LP and LP + LIV groups. We have previously established the multipotentiality of MSCs at EP timepoints^23,27,34–39^. In order to check the potential changes in basal mRNA expression at LP timepoints, mRNA was extracted from undifferentiated samples that kept in growth media for seven days and pooled mRNA was probed via Mouse Stem Cell RT² Profiler PCR Array (Qiagen, 330231 PAMM-405ZA). Shown in Table S1, mRNA expression of twenty genes in either the LP and the LP + LIV, including the ones related to cell cytoskeleton and focal adhesions such as Integrins alpha V, alpha X, beta 1 and Vimentin were changed more than 2-fold compared to P6 MSCs. Thirty eight genes in the LP and the LP + LIV groups showed less than 2-fold change in their mRNA expression when compared to P6. These thirty eight genes included RUNX2, PPARG, SOX9, SOX2, COL1A1, FGF2 and TGFB3 suggesting no large drifts in cell phenotype.

**Figure 4:**
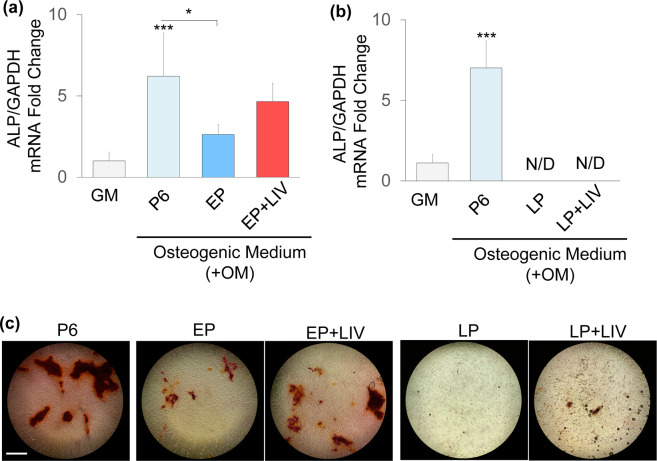
LIV improves osteogenesis at early passage. Alkaline phosphatase (ALP) mRNA expression was quantified and normalized to undifferentiated P6 MSCs kept in growth medium (i.e. GM group was set to 1). (**a**) Comparing to growth medium (GM), osteogenic induction increased *ALP* mRNA by 6.21-fold in P6 MSCs (p < 0.0001). EP MSCs had ALP mRNA expression of 2.62-fold and was significantly lower than osteogenic P6 control (p < 0.05). *ALP* expression of EP + LIV MSCs was not significantly different (−22%) than the osteogenic P6 control MSCs and remained 4.24-fold higher when compared to P6 MSCs kept in growth medium. (**b**) LP and LP + LIV groups had no detectable ALP mRNA while osteogenic P6 MSCs showed a 7-fold increase when compared to P6 MSCs kept in growth medium. (**c**) After 21 days of osteogenic induction P6, EP and EP + LIV groups were able to form visible calcium deposits. LP and the LP + LIV groups were unable to form visible calcium deposits. Experiments at EP and LP time points were independently run and analyzed. n = 3/grp, group comparisons were made using one-way ANOVA followed by a Newman-Keuls post-hoc test. p < 0.05, **p < 0.01, ***p < 0.001, against control and each other.

After seven days of osteogenic induction, alkaline phosphatase (*ALP*) mRNA expression was quantified and normalized to undifferentiated P6 MSCs kept in growth medium (i.e. GM group was set to 1). Experiments at EP and LP timepoints were independently run and analysed. Shown in Fig. 4a, osteogenic induction increased *ALP* mRNA by 6.21-fold in P6 MSCs (p < 0.0001). *ALP* expression of the EP MSCs was 2.62-fold higher than the *ALP* expression P6 MSCs kept in growth medium, a 58% decrease compared to osteogenic P6 controls (p < 0.05). *ALP* expression of EP + LIV MSCs was not significantly different (−22%) than the osteogenic P6 control MSCs and remained 4.24-fold higher when compared to P6 MSCs kept in growth medium (Fig. 4a). At later passages (Fig. 4b**)**, LP and LP + LIV groups had no detectable *ALP* mRNA while osteogenic P6 MSCs showed a 7-fold increase when compared to P6 MSCs kept in growth medium. As this suggested that LP MSCs are unable to undergo osteogenic differentiation, we next tested their ability to form calcium deposits after 21 days via Alizarin Red staining. As shown in Fig. 4c, P6, EP and EP + LIV groups were able to form calcium deposits. Corroborating *ALP* mRNA findings, both the LP and the LP + LIV groups were unable to form visible calcium deposits at day 21.

**Figure 5:**
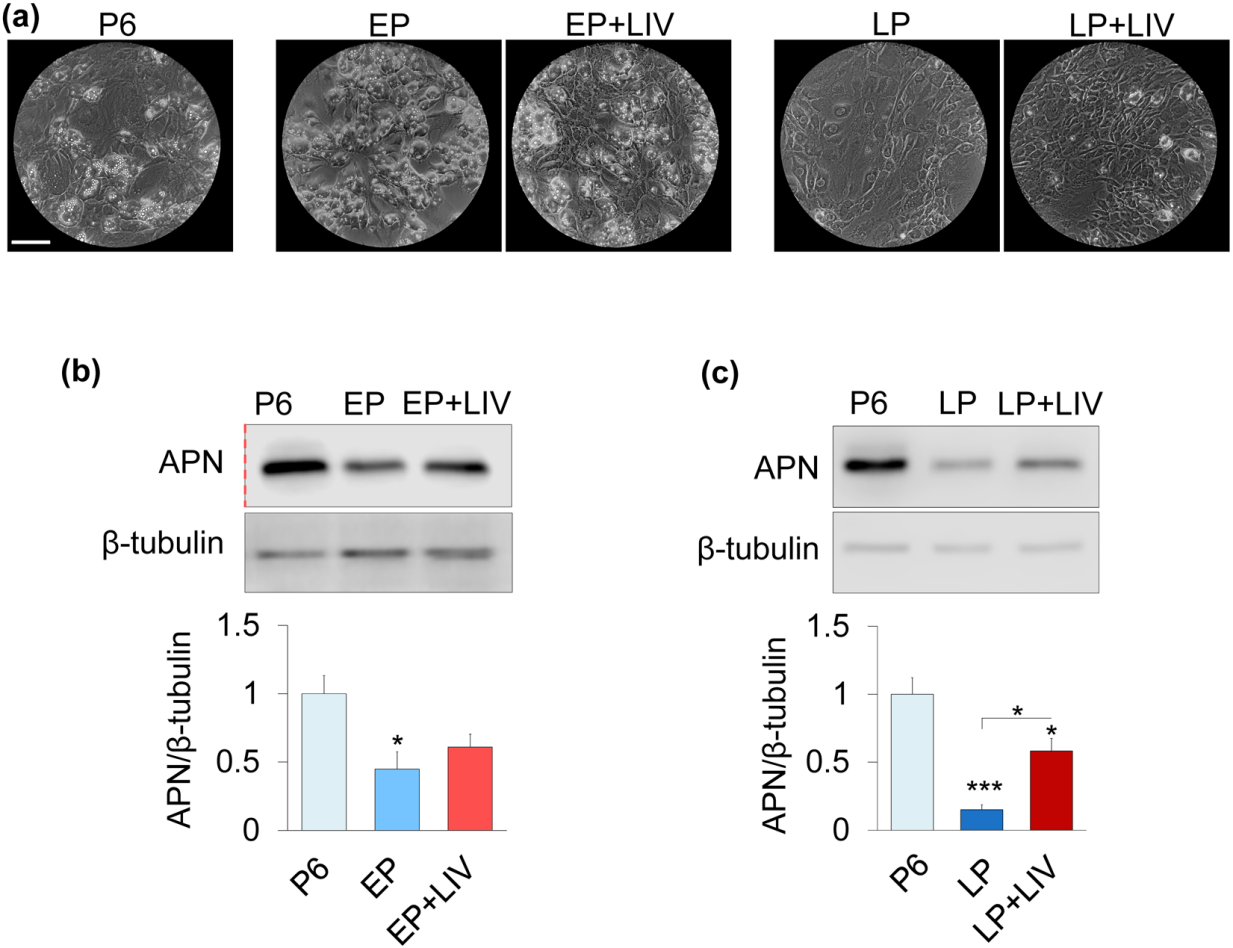
LIV improves adipogenic differentiation. (**a**) After 3 days of adipogenic induction, with the exception of LP, all groups were able form visible intracellular fat droplets. (**b**) APN protein levels were decreased 65.3% (p < 0.05) and 39.1% (NS) in the EP and EP + LIV groups, respectively. (**c**) The P6 group showed robust APN protein levels after three days, APN levels in LP was 85% lower when compared to P6 (p < 0.001). The LP + LIV group showed a 366% increased APN levels compared to the LP group (p < 0.05) while still remaining 42% lower than P6 control (p < 0.05). Experiments in (**b**,**c**) were performed and analyzed independently. Scale bar: 200 µm. n = 3/grp. Group comparisons were made using one-way ANOVA followed by a Newman-Keuls post-hoc test. p < 0.05, **p < 0.01, ***p < 0.001, against control and each other. Red dashed line in 5b indicates a cropped blot. Full-length blots are presented in Supplementary Fig.S2.

We next tested the adipogenic capacity. As shown in Fig. 5a, after three days of adipogenic induction, with the exception of the LP group, all groups were able to form visible intracellular fat droplets. Adipogenesis was further quantified by probing adiponectin (APN) protein levels via western blotting. All measurements were normalized to P6 (i.e P6 was set to 1) and experiments at EP and LP timepoints were independently run. As shown in Fig. 5b **and** Fig. 5c, the P6 controls showed a robust APN protein expression after three days. Normalized to P6, APN protein levels of the non-LIV EP group was reduced to 0.44, a 65.3% decrease (p < 0.05). APN protein levels of the EP + LIV group was also reduced to 0.6, a 40% decrease, but remained not-significantly different compared to P6 (Fig. 5b). Shown in Fig. 5c APN levels in the LP group was 0.15, 85% lower than the P6 control (p < 0.001). APN levels of the LP + LIV was 0.58, showing a 366% increase in compared to the LP group (p < 0.05), while still remaining 42% lower than the P6 group (p < 0.05).

### Observed changes in protein profiles during prolonged culture and long-term LIV application

**Figure 6:**
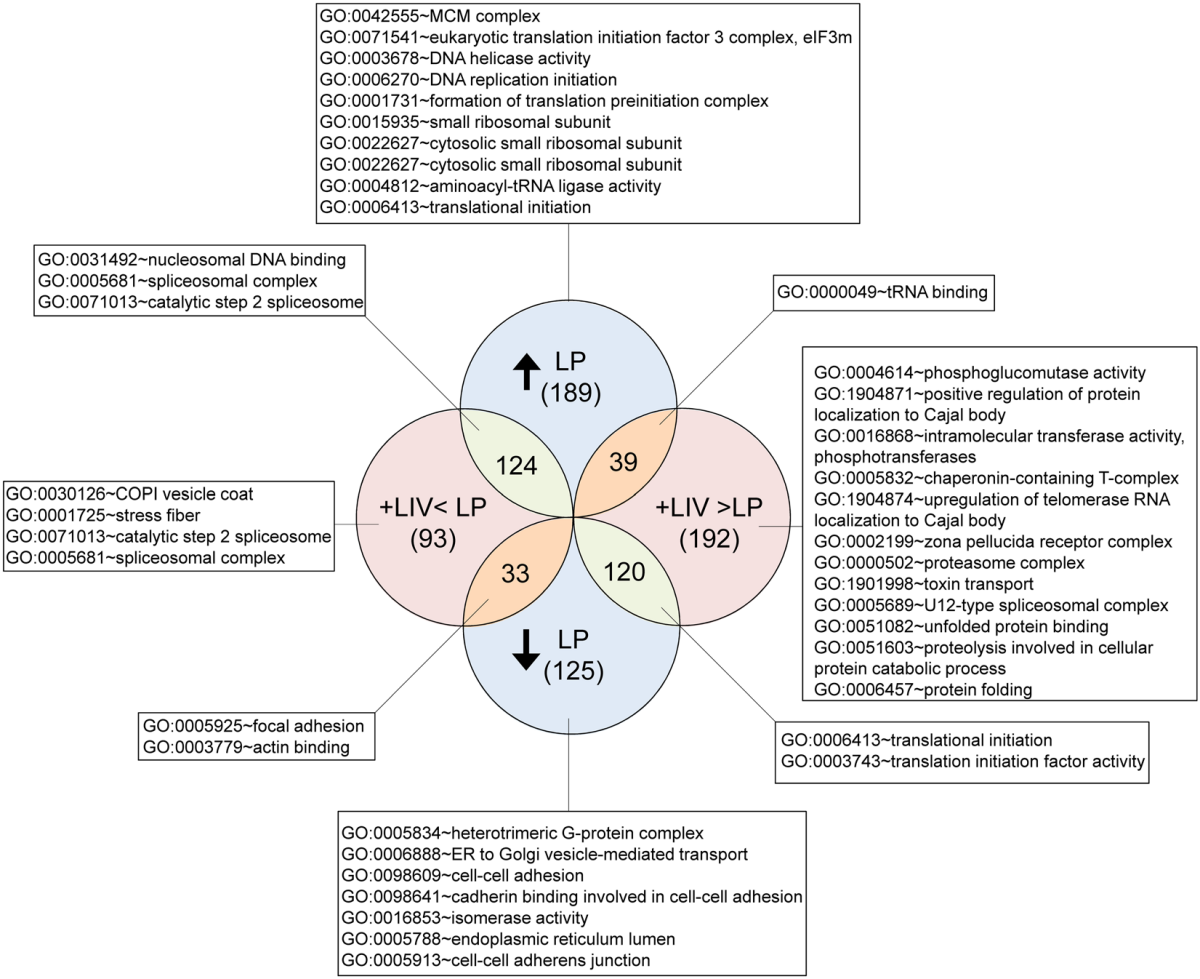
Changes in protein profiles during *in vitro* expansion and long-term LIV application. When compared to P6, total of 915 unique proteins were identified to be changed ±25% or more in LP and LP + LIV groups. Functional Annotation Clustering analysis was done via DAVID v6.8 (david.ncifcrf.gov) to identify GO TERMS associated with P6, LP and LP + LIV groups. Only terms with Benjamini score <0.05 and enrichment score of >10 were reported. n = 3/grp.

Since the largest differences in proliferation and differentiation were identified in the LP groups, we used tandem mass spectrometry to quantify the differential and synergistic changes in protein profiles during prolonged culture and long-term LIV treatment by comparing P6, LP and LP + LIV groups. When compared to P6, a total of 915 unique protein changes were identified in the LP and LP + LIV groups (±25% or more). Figure 6 shows the results of functional annotation clustering analysis of those 915 proteins with Benjamini score <0.05 and enrichment score of>10. The Full list of identified pathways with a Benjamini score <0.05 were presented in Table S2. As expected, this analysis yielded large number of interactions. Most of the identified pathways were related to DNA and RNA function as well as protein synthesis. Most notably, both the LP and LP + LIV groups showed changes in the protein levels associated with focal adhesions, actin binding, stress fibers, cell-cell adhesions and oxidoreductase activity pathways which informed our subsequent analysis.

### *In vitro* expansion and LIV synergistically decrease proteins associated with cytoskeleton and extracellular matrix

**Figure 7:**
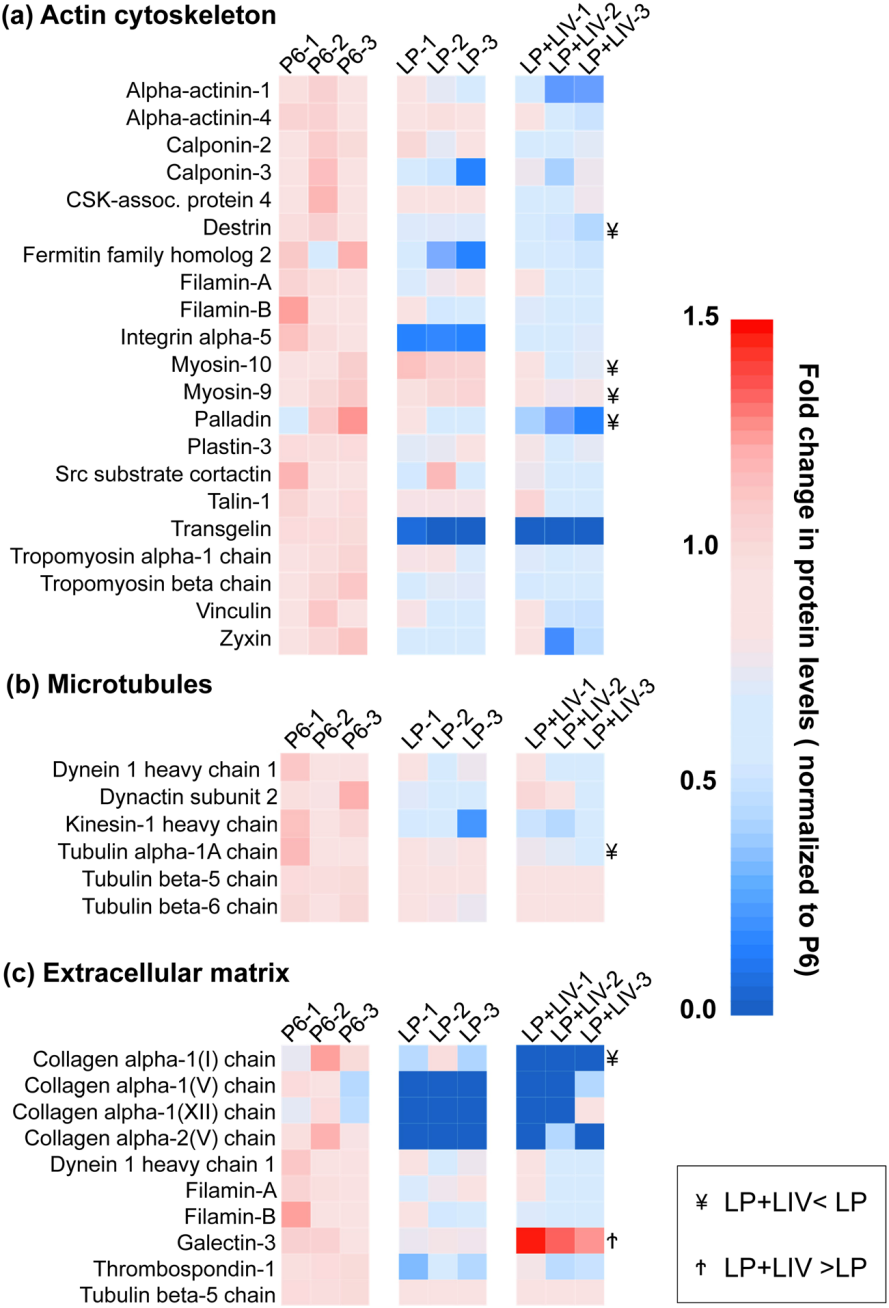
*In vitro* expansion and LIV synergistically decrease proteins associated with cytoskeleton and extracellular matrix. (**a**) 21 proteins relating to actin cytoskeleton were significantly decreased in either the LP or LP + LIV group. (**b**) Compared to P6, we have also identified six significantly downregulated microtubule related proteins, including three tubulin alpha and beta chains. (**c**) Extracellular matrix (ECM) related proteins, alpha-1 and/or apha-2 chains of Collagens I, V and XII were decreased by either LP or LP + LIV. n = 3/grp, group comparisons were made using one-way ANOVA followed by a Newman-Keuls post-hoc test. All presented proteins were p < 0.05 as identified via one-way ANOVA. Identified differences (p < 0.05) between LP and LP + LIV were further denoted as ¥: LP + LIV < LP, Ϯ: LP + LIV > LP.

As DAVID analysis identified changes in the focal adhesions, actin binding and stress fibers pathways, we further investigated the changes in the cytoskeleton related proteins in both the LP and LP + LIV groups, we used P6 MSCs as a baseline and only reported significantly changed proteins in either LP or LP + LIV MSCs (p < 0.05). Differences between the LP and LP + LIV were further denoted (¥: LP + LIV < LP, Ϯ: LP + LIV > LP). Findings represented in Fig. 7a showed significant decrease in 21 proteins related to actin cytoskeleton (p < 0.05). Notably, along with decreased levels of calponin-2 and calponin-3, protein levels of transgelin, an actin-crosslinking protein of the calponin family that has been shown to play a role in osteogenic and adiopogenic commitment of skeletal progenitors^40^, were depleted in both LP and LP + LIV groups. Protein levels of destrin, palladin, myosin-9 and myosin-10 remained lower in the LP + LIV group when compared to LP MSCs (¥, p < 0.05). Shown in Fig. 7b, we have also identified six downregulated microtubule related proteins, including three tubulin alpha and beta chains (p < 0.05). Tubulin alpha-1A chain was lower in the LP + LIV group when compared to the LP group (p < 0.05). Finally, quantifying extracellular matrix (ECM) related proteins in Fig. 7c, alpha-1 and apha-2 chains of collagens I, V and XII were decreased in both the LP and LP + LIV groups (p < 0.05). Compared to the LP group, collagen alpha-1(I) chain was significantly downregulated in the LP + LIV group (¥, p < 0.05) while galectin-3 levels were upregulated (Ϯ, p < 0.05).

### Mechanical activation of focal adhesion kinase was not altered by prolonged culture or LIV

**Figure 8:**
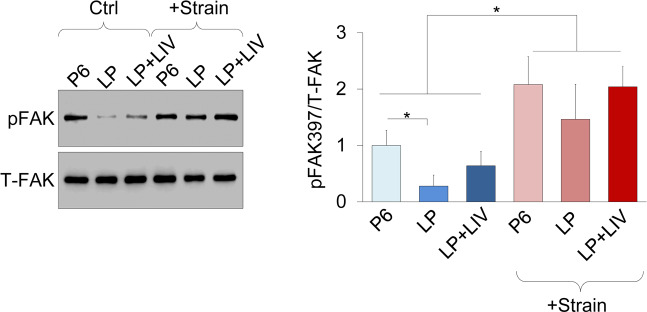
Mechanical activation of focal adhesion kinase was not altered by prolonged culture or LIV. MSCs were subjected to 2% equibiaxial strain at a rate of 0.2 Hz for 20 minutes and probed for Focal adhesion Kinase phosphorylation on Tyr397residue (pFAK). pFAK levels showed an 87% decrease (p < 0.05) in the LP group while LIV treated LP + LIV MSCs had a 300% increase in pFAK levels compared to the LP group. Upon strain application all three groups significantly increased pFAK levels compared to unstrained controls (p < 0.05) but were not statistically different from each other. n = 3/grp, group comparisons were made using one-way ANOVA followed by a Newman-Keuls post-hoc test. p < 0.05, **p < 0.01, ***p < 0.001, against control and each other. Full-length blots are presented in Supplementary Fig.S2.

As cytoskeleton, focal adhesion and ECM related proteins were decreased in prolonged culture and LIV treatment groups, we tested whether mechanical activation of focal adhesions were also altered compared to P6 MSCs. As we have reported previously, MSCs were subjected to 2% equibiaxial strain at a rate of 0.2 Hz for 20 minutes^39^. All measurements were normalized to P6 (i.e P6 was set to 1). Immediately after strain application, cells were probed for FAK phosphorylation on Tyr397 residue (pFAK), indicative of integrin engagement^41^. As shown in Fig. 8, baseline pFAK levels in the LP group was 0.27, decreasing 83% compared to P6 (p < 0.05). pFAK measurement of LIV treated LP + LIV MSCs was 0.63 which was not different when compared to P6 control MSCs. Upon strain application, all three groups experienced significant increases in pFAK levels compared to unstrained controls (p < 0.05) but were not statistically different from each other.

### Prolonged culture but not LIV decreases proteins associated with oxidoreductase activity

**Figure 9:**
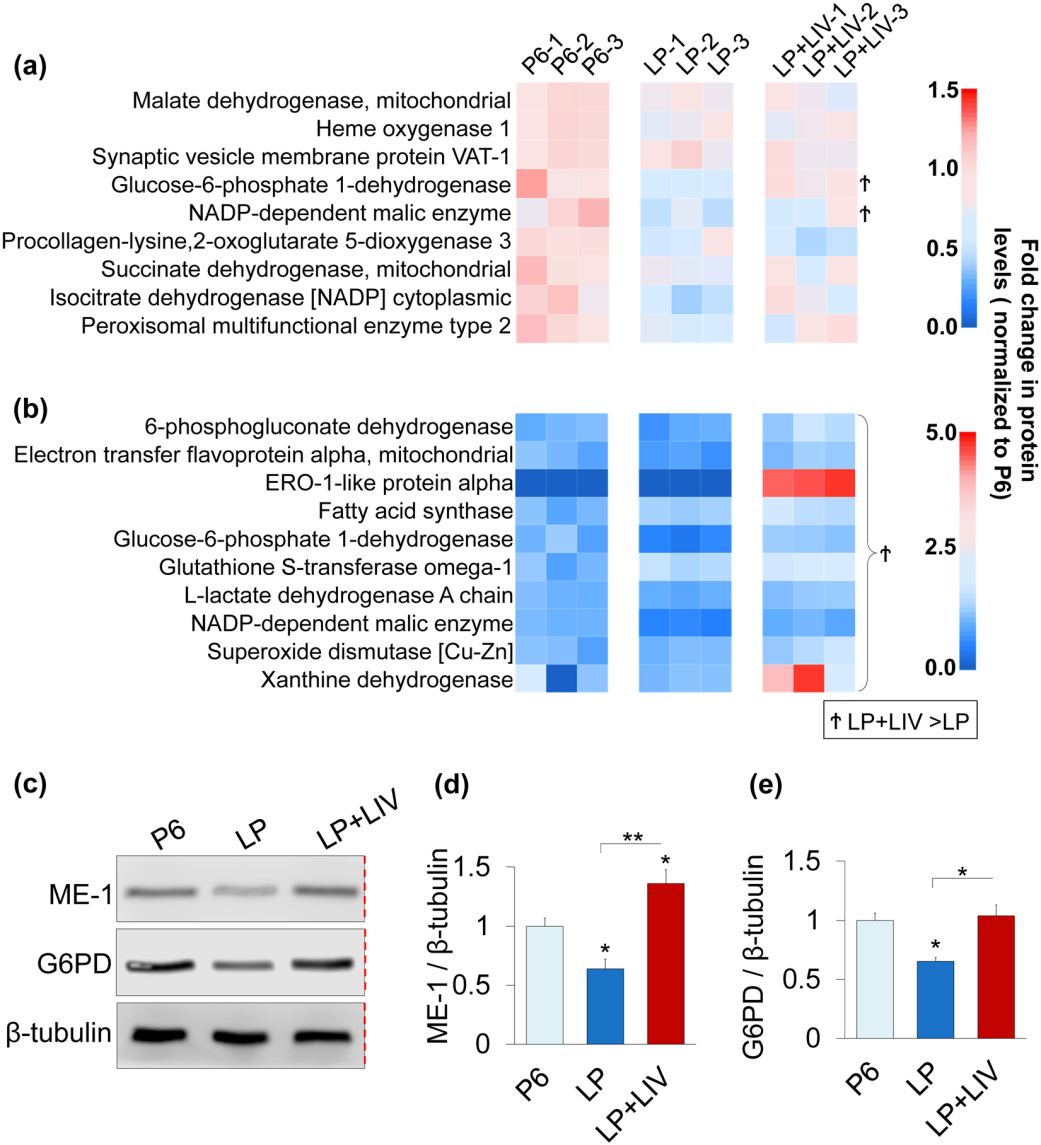
Prolonged culture but not LIV decreases proteins associated with oxidoreductase activity. (**a**) *In vitro* expansion of MSCs significantly decreased nine proteins related to oxidoreductase activity in LP cells (p < 0.05). G6PD and ME-1 were significantly upregulated in LP + LIV compared to LP (^Ϯ^p < 0.05) (**b**) Ten proteins associated with oxidoreductase activity exhibited significantly higher levels in the LP + LIV group compared to LP (p < 0.05) but were not changed compared to P6. Further, xanthine dehydrogenase and ERO1-like protein alpha were significantly elevated in the LP + LIV group when compared to the P6 group (p < 0.05). Identified differences (p < 0.05) between LP and LP + LIV were further denoted as Ϯ: LP + LIV > LP. (**c**) Representative western blots using primary antibodies against ME-1, G6PD and β-tubulin. Red dashed line indicates a cropped blot, full-length blots are presented in Supplementary Fig.S2. Samples derive from the same experiment and that blots were processed in parallel. (**d**) All measurements were normalized to P6 (i.e P6 was set to 1). Protein levels in the LP group was 0.63, a 37% decrease compared to the P6 group (p < 0.05). Average ME-1 protein level of the LP + LIV group was 1.36, a 36% (p < 0.05) and a 215% (p < 0.01) increase compared to the P6 and the LP groups. (**e**) Total G6PD protein level of the LP group was 0.65, remaining 35% lower compared to P6 (p < 0.05). G6PD levels of the LP + LIV group was back to 1.03 and was not significantly different than the P6 group, while remaining 58% larger than the LP group (p < 0.05). n = 3/grp. Group comparisons were made using one-way ANOVA followed by a Newman-Keuls post-hoc test. p < 0.05, **p < 0.01, ***p < 0.001, against control and each other.

ANOVA analysis in conjunction with DAVID pathway analysis revealed that *in vitro* expansion of MSCs significantly decreased nine proteins related to oxidoreductase activity in LP cells (p < 0.05, Fig. 9a). Notably, two proteins Glucose-6-phosphate 1-dehydrogenase (G6PD) and NADP-dependent malic enzyme (ME-1) were significantly upregulated in LP + LIV compared to LP (Ϯ, p < 0.05). As depletion of both ME-1^42^ and G6PD^43^ were associated with increased SA-βgal activity and decreased cell proliferation, we have further probed these two proteins via western blotting. All measurements were normalized to P6 (i.e P6 was set to 1). As shown in Fig. 9c–e, ME-1 protein levels in the LP group was 0.63, a 37% decrease compared to the P6 group (p < 0.05). Average ME-1 protein level of the LP + LIV group was 1.36, a 36% (p < 0.05) and a 215%(p < 0.01) increase compared to the P6 and the LP groups. Similarly, total G6PD protein level of the LP group was 0.65, remaining 35% lower compared to P6. (p < 0.05). G6PD levels of the LP + LIV group was back to 1.03 and was not significantly different than the P6 group, while remaining 58% larger than the LP group (p < 0.05). When we relaxed the proteomics search criterion by including genes that were not changed between the P6 – LP or P6 – LP + LIV groups, proteomics further identified 8 other proteins associated with oxidoreductase activity that exhibited significantly higher levels in the LP + LIV group compared to the LP group but were not changed compared to P6 (Ϯ, p < 0.05, Fig. 9b). Two of the largest changes were seen in the xanthine dehydrogenase and ERO-1-like protein alpha.

## Discussion

Exercise is a principal source of mechanical signals that are universally recognized to maintain a healthy musculoskeletal system. However, in the long-term, how mechanical components of exercise affect stem cells in the bone marrow is unknown. In this study we used an *in vitro* expansion model to test the effects of daily LIV application on primary mesenchymal stem cells up to 60 passages. LIV treated cells proliferated faster, averaging 27.7% higher doubling rates compared to non-LIV group (Fig. 2a). We recently reported that decreased cell proliferation under simulated microgravity results in decreased levels of RanGAP1 (Ran GTPase activating protein 1), RANG (Ran-specific GTPase-activating protein) and CDK1 (Cyclin-dependent kinase 1) proteins^27^. We have further reported increased RanGAP1 and RANG levels when LIV was used to rescue simulated microgravity-induced proliferation loss. While our analysis here did not reveal any cell cycle specific pathways, as shown in Table S2 the LP + LIV group showed increased protein levels of RanGAP1 (+72%, p < 0.001), RANG ( + 72%, p < 0.001) and CDK1 (62%, NS). It has been reported that RanGAP1 plays a role in the recruitment of CDK1 into the nucleus to mediate mitotic spindle assembly during cell cycle^44,45^, suggesting a possible role of LIV on cell cycle progression during both simulated microgravity and prolonged cell expansion.

Our finding suggest that LIV alters the number of SA-β-gal positive cells during prolonged passage but have no effect on other senescence associated markers p16 and telomere length. During early passages we found no difference in SA-β-gal staining, which suggests minimal changes in cell senescence. At later passages, LIV-treated MSCs showed an almost 2-fold decrease in the number of SA-β-gal positive cells (Fig. 3b). The number of SA-β-gal positive cells in the LP + LIV group however, was still more than double compared to P6 MSCs. These observed changes in SA-β-gal were not seen for p16, there were no measurable differences in the number of p16 positive nuclei (Fig. 3c-d) and p16 protein levels at later passages were unchanged compared to P6 (Fig. 3f). Further, we did not see any differences in telomere lengths (Fig. 3e) between P6, LP and LP + LIV MSCs. Although the causality between senescence and telomere shortening remains an open question^46^, telomere shortening of fibroblastic cells during serial culturing has been previously reported^47^. It is unclear why neither serial passaging nor LIV treatment did not result in any difference in telomere attrition in our model. Other studies utilizing mesenchymal stem cells in culture have reported both decreased^48^ and unchanged^49^ telomere lengths due to the inherent variability in measuring telomere lengths. Further, murine cells constitutively express telomerase^50^, making the telomere length comparison between groups more complicated. However, as shown in Fig. 2a, cell growth curves were not entirely flattened out but rather slowed down. Combined with the unchanged telomere lengths and p16 levels, it is possible that positive SA-βgal staining does not represent a truly senescent culture condition, and we may have ended our study too early. Interestingly, we have identified two proteins G6PD and ME-1 that were significantly decreased in the LP group compared to P6 and were upregulated in the LP + LIV groups (Fig. 9c-e). Depletion of both ME-1^42^ or G6PD^43^ were associated with increased SA-βgal activity. These findings suggest that changes in the oxidative reductase pathway may explain the reduced SA-βgal staining in LP + LIV groups. Regardless our findings are important to quantify the effects of long-term LIV application during *in vitro* expansion of MSCs.

When applied in conjunction with osteogenic constraints, LIV augments osteogenic differentiation^6^. Our findings here suggest that prior application of LIV is beneficial to subsequent osteogenic differentiation during early passages. While non-LIV EP MSCs showed a significant decrease in *ALP* mRNA after seven days, daily LIV application increased *ALP* mRNA to levels that were not significantly different from the control group (Fig. 4a). Corroborating our findings that application of LIV increases osteogenic potential during *in vitro* expansion, a prior study testing the combined effect of a week-long adipogenic induction with LIV treatment (0.15 g, 90 Hz) observed an increase in osteogenic potential in the +LIV groups^51^. At later passages, neither LP nor LP + LIV MSCs expressed any measurable *ALP* mRNA. Both LP and LP + LIV MSCs were not able to form calcium deposits (Fig. 4c). These findings suggest that long-term culture is detrimental to osteogenic potential with or without LIV. Similar loss of osteogenic potential under long term culture was reported in MSCs isolated from rats^32^. Potentially contributing to decreased osteogenesis, proteomic analysis showed loss of collagen isoforms in both the LP and LP + LIV groups (Fig. 7c). Collagens play essential roles during osteogenesis, and reduced levels as well as mutations of collagen cause bone diseases such as osteogenesis imperfecta^52^.

P6 and EP MSCs under adipogenic constraints robustly differentiated into adipocytes (Fig. 5). At earlier passages there was a slight decrease in adipogenic capacity as measured by APN levels in both EP and EP + LIV MSCs compared to P6 control but APN levels were not different between the EP and EP + LIV groups. At later passages, LP MSCs became rounder but did not form any visible fat droplets and APN levels were severely decreased by 85%. LP + LIV MSCs on the other hand showed some fat droplets and had measurably more APN compared to the LP group, suggesting a more robust adipogenic capacity. Our findings regarding adipogenic and osteogenic lineages at later passages are interesting because at the epigenetic level bone marrow derived MSCs used in this study were found to be more attuned to the osteogenic lineage when compared to adipogenic lineage^53^. This suggests that structural or otherwise changes in LIV treated cells may predispose these MSCs to adipogenesis. Notably, we have found an overall increase of protein levels related to oxidoerductase activity in +LIV groups at later passages. ERO-1-like protein alpha was increased the most in +LIV groups. ERO-1 has been shown to promote prolonged adiponectin secretion in murine 3T3-L1 pre-adipocytes^54^ which suggest that upregulated levels of oxidoreductase signaling related proteins in +LIV groups may partially explain the increased adipogenic potential in LP + LIV MSCs.

Structurally, MSCs at later passages displayed increased circularity and were enlarged in size when compared to EP timepoints. While the underlying mechanism of why cell area was decreased in EP and increased back up in LP cells is not clear, enlarged size of senescent cells has been reported^55,56^. As shown in Fig. 2e,b, P6 cells were larger but less circular compared to LP. Shown in Table S1, LP MSCs display a number of changes in the mRNA expression of integrin and focal adhesion related genes. As the integrin expression profile is a strong regulator how cells interact with extracellular matrix and can regulate cell shape^57^, it is possible that re-enlargement of cell size in the LP group was due to integrin-related changes. LP + LIV MSCs on the other hand, were less circular (Fig. 2e) and more spindle-like (Fig. 2b) showing similar features to P6 MSCs. Phalloidin staining however, showed no discernable differences in the F-actin structure except that LP + LIV MSCs displayed a smaller cell and nuclear area (Fig. 2d, Fig. S1a). Concomitantly, we have found an overall downregulation of proteins belonging to actin and microtubule regulation in both LP and LP + LIV MSCs. Measurable decreases in myosin-10 and myosin-9 were observed in the LP + LIV group when compared to the LP group. Loss of myosins may account for the differences in cell size by reducing the F-actin contractility^58,59^. Despite these differences in actin and microtubule regulatory proteins, we have found no differences in the strain-induced activation of FAK, which suggests that cell mechanosensitivity is not diminished by daily application of LIV or *in vitro* expansion of MSCs.

In summary, we have found that LIV augments the slowed down proliferation during *in vitro* expansion of MSCs up to sixty passages. While *in vitro* expansion resulted in decreased levels of the senescence marker SA-β-gal, no changes in p16 protein and telomere length were observed. At early passages LIV was pro-osteogenic and long term LIV was associated with increased adipogenic capacity. While LIV did not alleviate the reduction of actin and microtubule proteins found during *in vitro* expansion, we have found recovery of oxidative reductase related proteins in LIV treated MSCs. One limitation of our study is that *in vitro* expansion is not a very physiologically relevant model of aging. Despite this inherent limitation, to our knowledge this is the first study to look at the effect of long-term isolated mechanical challenge (in the form of LIV) on prolonged culturing conditions of primary mesenchymal stem cells. Quantified observations of LIV-induced effects therefore, will be important to inform future studies that target both underlying mechanisms of observed LIV effects and the role of long-term mechanical loading on chronological aging.

## Methods and Materials

### MSC isolation

Primary marrow-derived MSCs were isolated from 8–10 wk male C57BL/6 mice, and were prepared after Peister et al^60^. Tibial and femoral marrow were collected in RPMI-1640, 9% FBS, 9% HS, 100 μg/ml pen/strep and 12 μM L-glutamine. After 24 h, non-adherent cells were removed by washing with phosphate-buffered saline (PBS) and adherent cells cultured for 4 weeks. Passage 1 cells were collected after incubation with 0.25% trypsin/1 mM EDTA × 2 minutes, and re-plated in a single 175-cm^2^ flask. After 1–2 weeks, passage 2 cells were re-plated at 50 cells/cm^2^ in Iscove modified Dulbecco’s medium (IMDM) supplemented with 9% fetal bovine serum(FBS), 9% horse serum(HS), antibiotics, and L-glutamine. MSCs were re-plated every 1–2 weeks for two consecutive passages up to passage 5 and tested for osteogenic and adipogenic potential, and subsequently frozen. For experimental procedures, fetal calf serum (FCS) was obtained from Atlanta Biologicals (Atlanta, GA). Culture media, trypsin-EDTA, and antibiotics were supplied from Invitrogen (Carlsbad, CA). MSCs^61^ were maintained in IMDM with FCS (10%, v/v) and penicillin/streptomycin (100 μg/ml). All methods were carried out in accordance with relevant guidelines and regulations of Boise Institutional Animal Care and Use Committee and Institutional Biosafety Committee. All procedures were approved by Boise State University Institutional Animal Care and Use Committee, and Institutional Biosafety Committee.

#### Application of LIV

Vibrations were applied at room temperature using methods we previously established^23,27^. Briefly, LIV was applied twice for 20 min separated by a 2 h rest period.

#### *In vitro* expansion model

Passage 5 MSCs were plated into 75 cm^2^ flasks (Vwr, Radnor, PA) at a density of 1000 cell/cm^2^. Experimental groups were subjected to twice daily LIV regimen (0.7 g, 90 Hz, 20 min) outside of the incubator (+LIV). While control MSCs were taken out of the incubator for 20 minutes twice daily, they were not vibrated. Each day, flasks were observed for confluence and when either one of the groups reached 70% confluence (non-contact), both groups were trypsinized, counted and re-plated at the density of 1000 cell/cm^2^. This protocol was repeated until passage 60 (P60). The entire procedure took about 8 months. Schematic of experimental procedure is given in Fig. 1a. Between passages P12 and P15, in addition to serial culturing, MSCs were plated for battery of tests (Fig. 1b). All these assays were performed 24 h after plating with no LIV applied within the 24 h period. Data collected between P12 and P15 were designated as early passage (EP) and results were pooled for a total of n = 3 for each condition. Similarly, another battery of tests were performed between P57 and P60 and results were pooled as late passage (LP). Both EP and LP groups were compared against their +LIV counterparts. During each assay P6 cells were used as baseline controls. In order to compare EP or LP timepoints to P6, at any given assay P5 cells were thawed from frozen stock and cultured to P6 and compared to either EP or LP using independent experiments.

#### Determination of cell and nuclear size

Cells were plated into chamber slides (Ibidi µslide # 80421) at a density of 2000cells/cm^2^ and were fixed with 2% paraformaldehyde after 24 h. Nuclei and F-actin were stained per standard protocols using Hoechst 33342 (Life Technologies) and Alexa Fluor 488 conjugated Phalloidin (Life Technologies). Epifluorescent and phase contrast images were collected via an inverted microscope (Revolve, Echo Labs). Samples were collected randomly to reach N = 250/group. Further image processing and analyses were done using NIH imageJ software (https://imagej.nih.gov/ij/). Cell and nuclear area were quantified by using built-in functions and reported as pixel^2^. Partial cells and nuclei were excluded from analysis.

#### Senescence Associated β-galactosidase (SA-β-gal) staining

SA-β-gal staining was performed via senescence staining kit (#9860, Cell Signaling Technology, Danvers, MA). Cells were plated into slide chambers (# 80421, µslide, Ibidi, Germany) at a density of 2000 cell/cm^2^ and were fixed and stained after 24 h according to manufacturer instructions. After staining overnight, the staining solution was removed. 1X PBS was added to prevent drying. Bright field images were taken at 10x via an inverted microscope (Revolve, Echo Labs). 10 images were gathered per slide chamber. NIH ImageJ software were used to count SA-β-gal positive cells, which were defined as visibly blue stained cells with more than 50% of cell appeared blue and reported as percent of total cells counted. On average, N = 1350 cells per group were counted.

#### Telomere length

Cells were plated into 6-well plates (Corning, Corning, NY) at a density of 10000 cell/cm^2^ (N = 6/grp). In order to determine the relative telomere length in samples, genomic DNA was isolated using samples stored in DNAzol (Thermo Fisher Scientific, Grand Island, NY) via ethanol precipitation and resuspension in 8 mM NaOH. DNA was further purified using a Zymo DNA Clean and Concentrator kit (Zymo Research, Irvine, CA) and quantified using a Nanodrop (Thermo Fisher Scientific). For each sample, three qPCRs were be performed: the first one to amplify the telomeric DNA using primers^62^ and the second and third qPCRs to amplify two invariant copy number control genes to ultraconserved elements (UCE) developed by Hudon et al^63^. These provide an internal control to normalize the starting amount of DNA. Quantitative PCR (qPCR) was performed using a Roche LC96. PCR reactions were carried out in 20 µL volumes containing ~8 ng DNA, 10 µl of 2x Biotium Fast Plus EvaGreen qPCR Master Mix and 10 pmol each forward and reverse primers (500 nM final primer concentration). A standard curve for each primer pair was included in triplicate on each plate. The standard curve was prepared by seven serial dilutions (1∶5) of DNA. We used a two-step thermal cycling profile of 95 °C for 2 min, followed by 40 cycles of 95 °C for 5 s and 55 °C for 30 s, with signal acquisition at the end of the 55 °C step. The standard curve was used to transform Ct (cycle threshold) values to DNA concentration. All samples were run in triplicate and the mean used for subsequent calculations. The relative telomere length was reported by dividing the telomere DNA concentration by the averaged amount of the two UCE genes. A list of PCR primers used are given in Table S4.

#### Adipogenic and osteogenic differentiation assays

For adipogenic experiments, MSCs were plated at a density of 10,000 cell/well into 6 six-well dishes (Corning). The following day growth medium was replaced with adipogenic medium containing, α-MEM, 10 µM indomethacin, 0.1 µM dexamethasone and 5 µg/ml insulin. Cultures were incubated for three days. Adipocytes were identified via microscopy and total levels of the adipocyte specific protein adiponectin (APN) were quantified via western blotting as we have previously reported^23^. For osteogenic experiments MSCs were plated at 26,000 cells/cm^2^ in six-well dishes (Corning). The following day, IMDM media was replaced with an osteogenic media of α-MEM, ascorbic acid (50 μg/ml) and β-glycerophosphate (10 mM), which was changed every 48 hrs as we have previously reported^37^. P6 cells in growth medium served as a negative control. After seven days, osteogenesis was assessed via qPCR against alkaline phopsthatase (ALP) and formation of calcium nodules were confirmed via alizarin Red Staining after 21 days as we have previously reported^24^.

#### Strain experiments

Cells were plated onto 6-well Bioflex plates with silicone bottoms (Flexcell International, Hillsborough, NC). Uniform 2% biaxial strain was delivered at 10 cycles per minute for 20 minutes using the Flexcell FX-5000 system (Flexcell International, Hillsborough, NC). Controls were handled the same way but were not strained. Following strain, Focal Adhesion Kinase (FAK) phosphorylation at Tyr 397 site was measured as we have previously reported^39^.

#### Real-time RT-PCR

We have used previously established qPCR protocol for this study^23,27^. Briefly, total RNA was extracted via the RNeasy mini kit (Qiagen, Germany) per manufacturer’s instructions. cDNA was generated using 1 μg RNA in a total volume of 20 μL. Amplification reactions contained primers at 0.5 μM, deoxynucleotide triphosphates (0.2 mM), and 0.03 U Taq polymerase along with SYBR-green (Molecular Probes, Inc., Eugene, OR) at 1:150,000. mRNA expressions were compared using ΔΔCt method as we previously reported^24^. PCR products from all species were run in triplicates and were normalized against GAPDH, which we previously used GAPDH as a reference gene inMSCs^24^. A list of primers were provided in Table S4. We have additionally used Mouse Stem Cell RT² Profiler PCR Array (Qiagen, 330231 PAMM-405ZA) via manufacturer instructions.

#### Western blotting

We performed western blotting using methods we previously described^23,26,27,64^. Briefly re-iterating the method, cells were lysed in RIPA (150 mM NaCl, 50 mM Tris HCl, 1 mM EDTA, 0.24% sodium deoxycholate,1% Igepal, adjusted to pH 7.5) supplemented with NaF (25 mM), Na3VO4 (2 mM), aprotinin, leupeptin, pepstatin, and phenylmethylsulfonylfluoride (PMSF) to avoid sample degradation. 20 μg protein from each sample was run on 9% polyacrylamide gels and transferred to polyvinylidene difluoride (PVDF) membranes. Membranes were blocked with 5%, w/v milk diluted in Tris-buffered saline containing Tween20 (TBS-T, 0.05%). Appropriate primary antibodies were then added and blots were incubated overnight at 4 °C with. After 12 to 24 h, samples were washed and incubated in horseradish peroxidase-conjugated secondary antibodies were added which were diluted 1: 5,000 (Cell Signaling). ECL plus (Amersham Biosciences, Piscataway, NJ) kit was used to detect chemiluminescence. Minimum of three independent experiments were analyzed to obtain densitometry using NIH ImageJ software. Primary antibodies used in the study are given in Table S4.

### Liquid chromatography – tandem mass spectrometry (LC-MS/MS) Based Proteomic Analysis

Proteomic analysis was performed as we previously reported^27^. In briefly reiterating our protocol, 20 μg of total protein from each sample was digested with trypsin/lys C mix (Promega, Madison, WI). Cell lystates were then precipitated with acetone and pellets were resuspended in 8 M urea which was reduced by dithiothreitol, and alkylated by iodoacetamide. Samples were diluted to 1 M urea concentration and were further incubated with trypsin/lys C overnight at 30 °C, desalted and purified using a reverse-phase C18 spin column. Resulting peptides were chromatographically separated on a reverse-phase C18 column (10 cm ×75 µm, 3 µm, 120 Å) and analyzed on a Velos Pro Dual-Pressure Linear Ion Trap mass spectrometer (Thermo Fisher Scientific) as described previously^65^.

Protein identification and quantification were done via Sequest HT algorithms in Proteome Discoverer 1.4 informatics software (Thermo Fisher Scientific). Swiss-Prot protein sequence database for mouse was available from www.unipro.org (une 15, 2018) which was utilized in the Sequest HT spectrum. Search parameters included: trypsin, maximum missed cleavage site of two, precursor mass tolerance of 1.5 Da, fragment mass tolerance of 0.8 Da, static modification of cysteine carbamidomethylation (+57.021 Da), and dynamic modification of methionine oxidation (+15.995 Da). False discovery rate (FDR) was calculated via a decoy database search. Proteins with one or more peptides (FDR < 0.05) were reported. Peptide spectrum matches (PSMs) for each protein calculated and reported for each protein. We further normalized protein read in each sample to total PSMs of the sample x 10,000. Following mass spectrometry, functional annotation clustering analysis was done using DAVID v6.8 (the Database for Annotation, Visualization and Integrated Discovery, david.ncifcrf.gov) to identify Gene Ontology (GO) terms. Medium classification stringency (default) was used.

### Statistical analysis

Results are presented as mean ± SEM unless indicated in figure legends. Densitometry and other analyses were performed on at least three independent experiments. As we previously reported^26,27^, for comparisons regarding qPCR or western blot data, differences between treatments within each biological replicate were assumed to follow a normal distribution due to large mean sample size (20,000 or more cells/group), thus for these comparisons, we have used two-tailed un-paired one way ANOVA followed by Newman-Keuls post-hoc tests. For other comparisons with smaller sample size, including proliferation, cell morphology and imaging we assessed normality Kolmogorov-Simirnov test (α = 0.05). For non-normally distributed data, we used non-parametric two-tailed Mann-Whitney U-test or Kruskal-Wallis test as indicated in figure legends. P-values of less than 0.05 were considered significant.

## Supplementary information

Supplementary Information Table S2.

Supplementary Information Table S3.

Supplementary Information Figures S1-S2, Tables S1, S4-S7

## Data Availability

The datasets generated and/or analyzed during the current study are available from the corresponding author on reasonable request.
