# Supplementary material for: Low Intensity Vibrations Augment Mesenchymal Stem Cell Proliferation and Differentiation Capacity during in vitro Expansion
Source: Sci Rep. 2020 Jun 10;10:9369. doi: 10.1038/s41598-020-66055-0 (PMC7286897; doi:10.1038/s41598-020-66055-0)
Supplement: Supplementary file 3 — Supplementary Information Figures S1-S2, Tables S1, S4-S7 [file 41598_2020_66055_MOESM3_ESM.pdf]

# Supplementary Information

## Low Intensity Vibrations Augment Mesenchymal Stem Cell Proliferation and Differentiation Capacity during *in vitro* Expansion

Bas G[1], Loisate S[1], Woods K[2], Hudon SF[2,3], Hayden EJ[3], Pu X[4], Beard R[4], Oxford JT[3,4], Uzer G[1†]

[1]Mechanical and Biomedical Engineering, Boise State University
[2] Biomolecular Sciences Graduate Program, Boise State University
[3] Biological Sciences, Boise State University
[4] Biomolecular Research Center, Boise State University
**† Corresponding Author**

**Funding support:**
NASA ISGC NNX15AI04H, NIH R01AG059923, 5P2CHD086843-03, P20GM109095, and P20GM103408

**Figure S1**

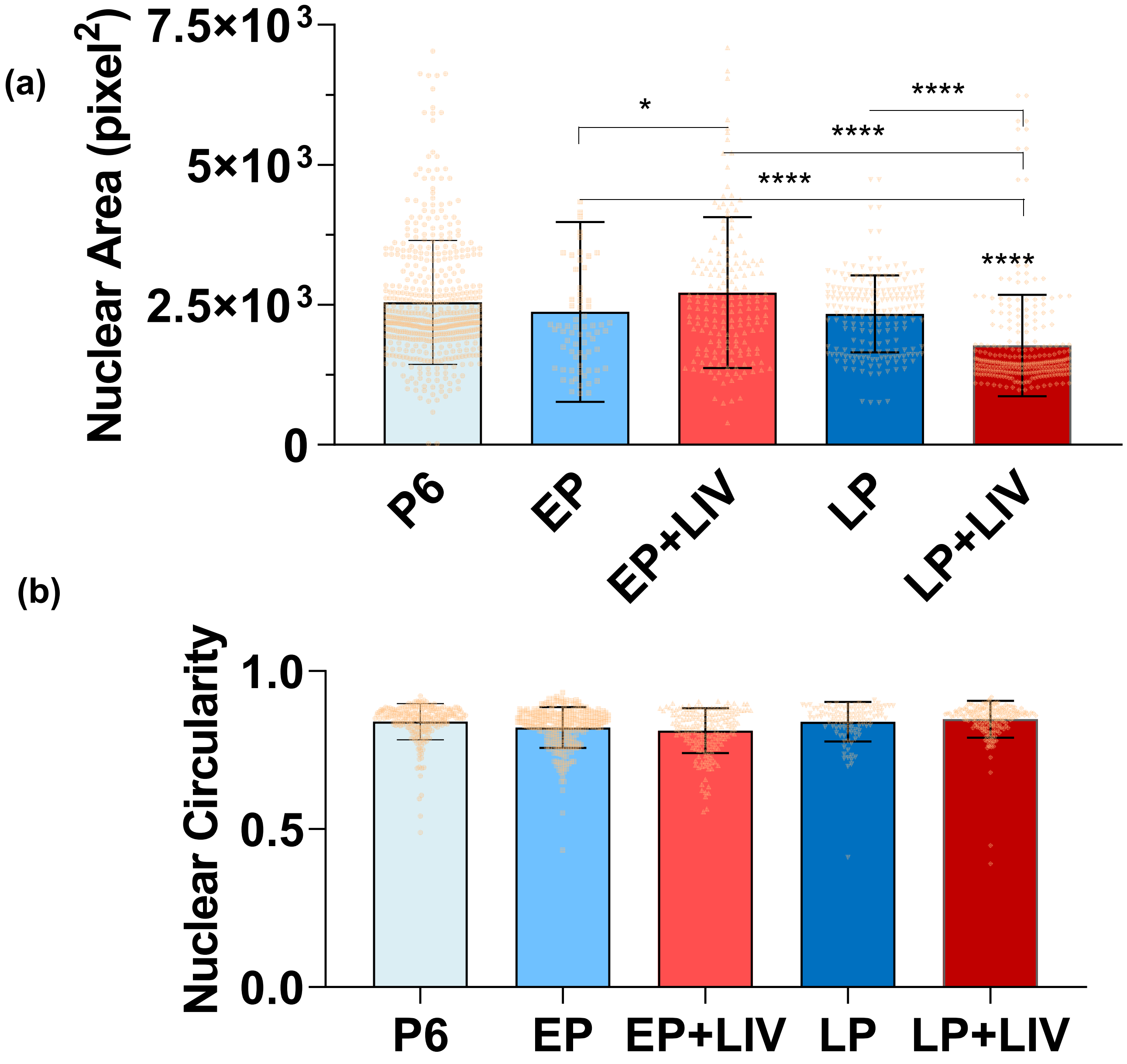


**Figure S1. a** nuclear areas of the EP, EP+LIV and the LP groups were not significantly altered when compared to the nuclear area of P6 MSCs which was $2.54 \times 10^3$ $px^2$. The nuclear area of the LP+LIV group was $1.77 \times 10^3$ $px^2$, exhibiting a 40% decrease compared to the P6 group. Nuclear area of the LP+LIV group also remained significantly lower than the EP, EP+LIV and the LP groups ($p<0.0001$). **b)** Nuclear circularity was not affected. Group comparisons were made using Kruskal-Wallis test followed by Dunn's multiple comparison test. $p<0.05$, ** $p<0.01$, *** $p<0.001$, **** $p<0.0001$against control and each other. Results are presented as mean ± STD.

**Table S1:** mRNA expression of P6, LP and LP+LIV groups after 7-days in growth media

| Description | Symbol | P6 | LP | LP+LIV |
|---|---|---|---|---|
| **mRNA expression (> ± 2-fold)** | | | | |
| ATP-binding cassette, sub-family B (MDR/TAP), member 1 | ABCB1 | 1.00 | 3.54 | 4.28 |
| Thy-1 cell surface antigen | THY1 | 1.00 | 3.03 | 2.40 |
| Vimentin | VIM | 1.00 | 2.63 | 1.10 |
| T-box 5 | TBX5 | 1.00 | 2.38 | 1.80 |
| K(lysine) acetyltransferase 2B | KAT2B | 1.00 | 0.37 | 0.13 |
| Interleukin 6 (interferon, beta 2) | IL6 | 1.00 | 0.32 | 3.02 |
| Activated leukocyte cell adhesion molecule | ALCAM | 1.00 | 0.27 | 0.10 |
| Tumor necrosis factor | TNF | 1.00 | 0.25 | 0.50 |
| Integrin, alpha V (vitronectin receptor, alpha polypeptide, antigen CD51) | ITGAV | 1.00 | 0.24 | 0.52 |
| Fucosyltransferase 1 (galactoside 2-alpha-L-fucosyltransferase, H blood group) | FUT1 | 1.00 | 0.21 | 0.42 |
| Nestin | NES | 1.00 | 0.15 | 0.04 |
| Ras homolog gene family, member A | RHOA | 1.00 | 0.13 | 0.12 |
| Integrin, alpha X (complement component 3 receptor 4 subunit) | ITGAX | 1.00 | 0.07 | 0.04 |
| CD44 molecule (Indian blood group) | CD44 | 1.00 | 0.05 | 1.35 |
| PTK2 protein tyrosine kinase 2 | PTK2 | 1.00 | 0.05 | 0.10 |
| KIT ligand | KITLG | 1.00 | 0.03 | 0.04 |
| Similar to histone deacetylase 1 | LOC487309 | 1.00 | 1.04 | 2.69 |
| Integrin, beta 1 (fibronectin receptor, beta polypeptide, antigen CD29 includes MDF2, MSK12) | ITGB1 | 1.00 | 1.34 | 2.41 |
| Prominin 1 | PROM1 | 1.00 | 1.72 | 2.36 |
| HNF1 homeobox A | HNF1A | 1.00 | 1.24 | 0.30 |
| Vascular endothelial growth factor A | VEGFA | 1.00 | 0.52 | 0.28 |
| **mRNA expression (< ± 2-fold)** | | | | |
| Fibroblast growth factor 2 (basic) | FGF2 | 1.00 | 1.28 | 1.97 |
| Intercellular adhesion molecule 1 | ICAM1 | 1.00 | 1.66 | 1.88 |
| Protein tyrosine phosphatase, receptor type, C | PTPRC | 1.00 | 0.62 | 1.68 |
| Phosphatidylinositol glycan anchor biosynthesis, class S | PIGS | 1.00 | 1.50 | 1.53 |
| Transforming growth factor, beta 3 | TGFB3 | 1.00 | 1.05 | 1.46 |
| SMAD family member 4 | SMAD4 | 1.00 | 0.89 | 1.39 |
| Runt-related transcription factor 2 | RUNX2 | 1.00 | 0.99 | 1.38 |
| Collagen, type I, alpha 1 | COL1A1 | 1.00 | 0.84 | 1.37 |
| POU class 5 homeobox 1 | POU5F1 | 1.00 | 1.02 | 1.37 |
| Solute carrier family 17 (anion/sugar transporter), member 5 | SLC17A5 | 1.00 | 1.20 | 1.30 |
| Nudix (nucleoside diphosphate linked moiety X)-type motif 6 | NUDT6 | 1.00 | 1.71 | 1.27 |
| Nerve growth factor receptor | NGFR | 1.00 | 1.61 | 1.24 |
| SMAD specific E3 ubiquitin protein ligase 2 | SMURF2 | 1.00 | 1.14 | 1.24 |
| Leukemia inhibitory factor (cholinergic differentiation factor) | LIF | 1.00 | 1.47 | 1.24 |
| Jagged 1 | JAG1 | 1.00 | 1.19 | 1.22 |
| Notch 1 | NOTCH1 | 1.00 | 0.52 | 1.14 |
| Similar to histone aminotransferase 1 (predicted) | LOC478799 | 1.00 | 0.84 | 1.13 |
| Insulin receptor | INSR | 1.00 | 1.65 | 1.12 |
| Transforming growth factor, beta 1 | TGFB1 | 1.00 | 0.87 | 1.12 |
| Bone gamma-carboxyglutamate (gla) protein | BGLAP | 1.00 | 1.38 | 1.11 |
| Lipoprotein lipase | LPL | 1.00 | 1.15 | 1.11 |
| Matrix metallopeptidase 2 (gelatinase A, 72kDa gelatinase, 72kDa type IV collagenase) | MMP2 | 1.00 | 0.59 | 1.08 |
| Microphthalmia-associated transcription factor | MITF | 1.00 | 0.68 | 1.01 |
| SMAD specific E3 ubiquitin protein ligase 1 | SMURF1 | 1.00 | 1.03 | 0.89 |
| 5'-nucleotidase, ecto (CD73) | NT5E | 1.00 | 1.16 | 0.87 |
| Kinase insert domain receptor (a type III receptor tyrosine kinase) | KDR | 1.00 | 1.39 | 0.84 |
| Wingless-type MMTV integration site family, member 3A | WNT3A | 1.00 | 1.21 | 0.82 |
| Insulin | INS | 1.00 | 1.24 | 0.73 |
| SRY (sex determining region Y)-box 9 | SOX9 | 1.00 | 0.50 | 0.67 |
| Platelet-derived growth factor receptor, beta polypeptide | PDGFRB | 1.00 | 1.78 | 0.66 |
| Vascular cell adhesion molecule 1 | VCAM1 | 1.00 | 1.11 | 0.62 |
| Von Willebrand factor | VWF | 1.00 | 0.55 | 0.59 |
| Melanoma cell adhesion molecule | MCAM | 1.00 | 1.45 | 0.58 |
| SRY (sex determining region Y)-box 2 | SOX2 | 1.00 | 0.59 | 0.53 |
| Peroxisome proliferator-activated receptor gamma | PPARG | 1.00 | 1.02 | 1.15 |

**Table.S4:** PCR Primers used

| PCR primers | |
|---|---|
| ALP -Forward | 5′-AACCCAGACACAAGCATTCC-3′ |
| ALP -Reverse | 5′-GCCTTTGAGGTTTTTGGTCA-3′ |
| GAPDH -Forward | 5′-ACCCAGAAGACTGTGGATGG-3′ |
| GAPDH -Reverse | 5′-CACATTGGGGGTAGGAACAC-3′ |
| UCE.359 - Forward | 5′-ATCTGAGACTTGTGACAT-3′ |
| UCE.359 -Reverse | 5′-GTGTTAATTGGTAATGACTATT-3′ |
| UCE.28 -Forward | 5′-AAATACCACCCAACAGTT-3′ |
| UCE.28 -Reverse | 5′-AAGCCCTATACAGATGGAT-3′ |
| Telomere (tel 1b) -Forward | 5′-CGGTTTGTTTGGGTTTGGGTTTGGGTTTGGGTTTGGGTT-3′ |
| Telomere (tel 2b) -Reverse | 5′-GGCTTGCCTTACCCTTACCCTTACCCTTACCCTTACCCT-3′ |

**Table.S5 :** Antibodies used and their final concentrations for western blots

| Antibodies | | Final Concentration |
|---|---|---|
| p-FAK Tyr397 (3283) | Cell Signaling | 1/1000 |
| FAK (sc-558) | Santa Cruz Biotechnology | 1/500 |
| Beta Tubulin (D3U1W) | Cell Signaling | 1/1000 |
| Adiponectin (C45B10) | Cell Signaling | 1/500 |
| ME1 (C-6) | Santa Cruz Biotechnology | 1/500 |
| P16 INK4A (D7C1M) | Santa Cruz Biotechnology | 1/500 |

**Table.S6:** Immunostaining antibodies and reagents and their final concentrations

| Immunostaining antibodies and Staining Reagents | | Final Concentration |
|---|---|---|
| Hoechst 33342 | Invitrogen | 1 µg/mL |
| Alexa Fluor 488 Phalloidin | Life Technologies | 0.1µM |
| Alizarin Red S | Sigma | 40mM |

**Table.S7 :** Cell Culture and Pharmacological Reagents

| Cell Culture and Pharmacological Reagents | | Final Concentration |
|---|---|---|
| IMDM | GIBCO | - |
| alpha-MEM | GIBCO | |
| FCS | Atlanta Biologicals | 10% v/v |
| Penicillin/streptomycin | GIBCO | 1% v/v |
| Insulin | SIGMA | 5ug/mL |
| Dexamethasone | SIGMA | 0.1uM |
| Indomethacin | SIGMA | 50uM |
| Beta-Glycerophosphate | SIGMA | 10mM |
| Ascorbic Acid | SIGMA | 50ug/uL |

# Figure S2

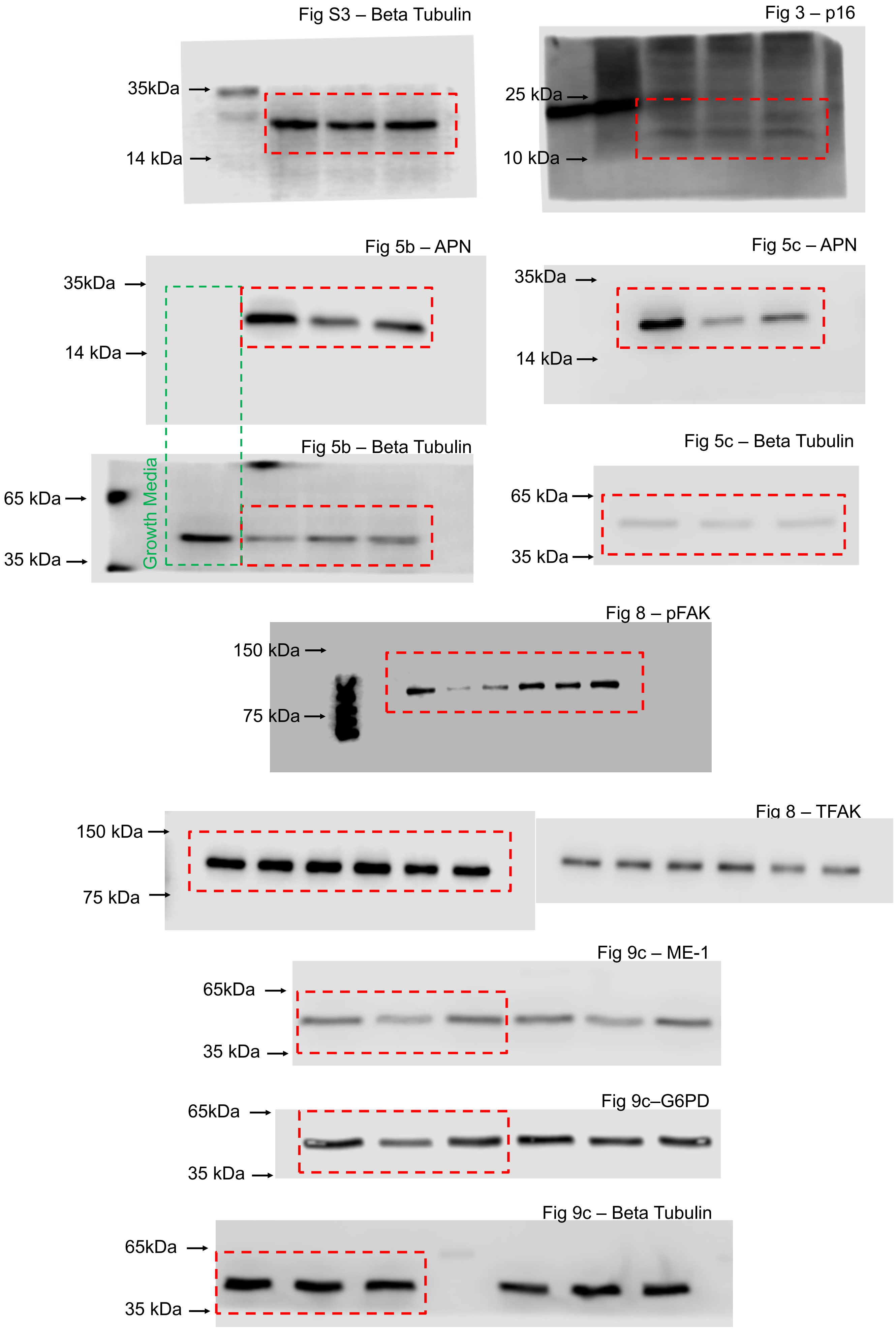


**Figure S2.** Unprocessed blots as obtained by LiCor C-DiGit blot scanner.
